# Lower CXCR3 expression in both patients with neovascular AMD and advanced stages of chronic myeloproliferative blood cancers

**DOI:** 10.1371/journal.pone.0269960

**Published:** 2022-06-16

**Authors:** Charlotte Liisborg, Vibe Skov, Lasse Kjær, Hans Carl Hasselbalch, Torben Lykke Sørensen

**Affiliations:** 1 Department of Ophthalmology, Zealand University Hospital, Roskilde, Denmark; 2 Faculty of Health and Medical Sciences, University of Copenhagen, Copenhagen, Denmark; 3 Department of Hematology, Zealand University Hospital, Roskilde, Denmark; Qatar University, QATAR

## Abstract

**Purpose:**

Peripheral T cell CXCR3 expression has been found uniquely lower in patients having neovascular age-related macular degeneration (nAMD) than in healthy individuals. The CXCR3-axis has been shown to have angiostatic and antifibrotic properties. We have recently investigated systemic markers in patients with myeloproliferative neoplasms (MPNs) because of their higher prevalence of AMD, and we have observed higher systemic chronic low-grade inflammation and immunosenescence signs in MPNs with drusen (MPNd) compared to those with normal retinas (MPNn). The MPNs evolve in a biological continuum from early cancer-stages (essential thrombocytosis, polycythemia vera) to the advanced myelofibrosis stage. Especially myelofibrosis is characterized by bone marrow angiogenesis and fibrosis, similarly to retinal observations in nAMD. We speculate if we can find lower CXCR3 expression in MPNs, particularly myelofibrosis and if differences are seen between MPNd and MPNn. We also wanted to compare expression in nAMD and intermediate (i)AMD.

**Methods:**

Patients in this cross-sectional study were 29 nAMD, 28 iAMD, 35 MPNd, and 27 MPNn. We performed flowcytometry on blood to measure CXCR3 expression.

**Results:**

CD8+CXCR3 expression in nAMD was 6,1%, significantly lower than in iAMD 16%, MPNd 11%, MPNn 12% (p-values<0.05). Similar results were seen for CD4+CXCR3 expression. We also found CXCR3 expression decreasing over the MPN-continuum. For instance, in myelofibrosis, intermediate monocytes expression was 6.2%, significantly lower than 18% in ET and 18% in PV (p-values<0.05).

**Conclusions:**

We find CXCR3 downregulation on T-cells and some monocyte subset in nAMD compared to iAMD, MPNd, and MPNn, in line with previous nAMD studies. We also find CXCR3 downregulation in most monocyte subsets over the MPN continuum. Systemic leukocyte CXCR3 expression could both be involved in changes seen in the retina and the bone marrow. Further understanding the CXCR3-axis in AMD and MPNs may elucidate underlying pathogenic mechanisms and reveal new targets for treatment.

## Introduction

Age-related macular degeneration (AMD) is a disease that affects the photoreceptors (PR), the retinal pigment epithelium (RPE), Bruch’s membrane (BM), and the choroid [1]. The disease stages are divided into early-, intermediate (iAMD)- and late AMD [2]. All AMD stages are characterized by extracellular deposits (drusen) accumulation below or above the RPE. The vision-threatening late-AMD stages are divided into two clinical entities, the often more slowly evolving geographic atrophy and the faster developing neovascular AMD (nAMD). In nAMD, angiogenesis as choroidal neovascularization (CNV) is a central feature. The abnormal blood vessel growth that seems to be driven significantly by vascular endothelial growth factor (VEGF) results in fragile vessels that leak fluid and blood into the retina with devastating visual consequences [1, 3]. Eventually, fibrosis arises from the pre-existing CNV lesions [4].

Angiogenesis is a normal physiological process needed, for instance, in wound healing and tissue repair, but it can also be an outcome of pathological conditions such as in nAMD, chronic inflammation, cancer, and ischemia. Pro- and anti-angiogenic factors control vascular homeostasis. Inflammation and angiogenesis are interconnected and involved in many pathological conditions, with cytokines as regulators of these processes, exerting effects on receptors shared by many cells throughout the body, including leukocytes and endothelial cells [5–7]. Chemokines are chemoattractant cytokines, small signaling proteins with diverse functions secreted by cells. The most studied function is cell migration of leukocytes, but chemokines have numerous activities and work on several cell types. For instance, chemokines and their receptors play a role in the immune cell activation and differentiation and thus the inflammatory process [8]. In addition, many members of the CC- and CXC- families of chemokines and their corresponding receptors have angiogenic and angiostatic properties. The interferon (IFN)-γ inducible chemokines CXCL9, CXCL10, and CXCL11 are strongly associated with Th1 mediated immune responses [9] and are also potent angiogenesis inhibitors functioning through the chemokine receptor 3 (CXCR3). CXCR3 is therefore involved in differentiating naïve T cells into Th1 effector cells and plays an important role in T cell function and trafficking [10]. Many chemokines also “cross-talk” with the pro-angiogenic VEGF, for instance, CXCL10-mediated inhibition of VEGF [11]. In summary, chemokines are key players in innate and adaptive immunity both in health and disease, and given the association between inflammation and the CXCR3-axis, it may not be unexpected that this system plays a role in many autoimmune diseases [12–14].

We have previously shown a significantly lower CD8+ T cell CXCR3 expression in patients with nAMD than in age-matched controls [15, 16]. Also, CXCL10 has been found elevated in the retinas of patients with AMD, and choroidal endothelial cells express its receptor CXCR3 [17]. Further, mice with genetic ablation of CXCR3 show exacerbated CNV formation [18].

We have recently studied systemic alterations in patients with myeloproliferative neoplasms (MPNs) because of their increased drusen and AMD prevalence [19]. The MPNs evolve in a biological continuum from essential thrombocythemia (ET) to polycythemia vera (PV) to the advanced myelofibrosis stage (MF). The earlier stages (ET & PV) are characterized by excess production of different myeloid terminally differentiated blood cell quantities, while the late MF stage show variable platelet and leukocyte counts and even anemia. Angiogenesis and bone marrow fibrosis are less pronounced in ET and PV but are essential in MF pathogenesis [20, 21]. We have reported, in MPNs, an association between drusen presence and chronic low-grade inflammation and drusen presence and signs of an aging immune system (immunosenescence). Patients with nAMD and MPNs share several characteristics, such as angiogenesis, fibrosis, and inflammation being involved in the pathogeneses.

Because CXCR3 is found to be uniquely lower in patients with nAMD, and this receptor has been shown to have anti-angiogenic and antifibrotic properties, the research questions/purposes for this study were: 1) Can we repeat our finding of low CXCR3 expression on leukocytes in patients with nAMD when comparing to iAMD? 2) since the MPNs are a biological continuum, can we find low CXCR3 expression in patients with dominant angiogenic and fibrotic stages of disease (which nAMD also could be considered as)? 3) We also wanted to compare the CXCR3 expression in nAMD to MPNs and measure the ligands of CXCR3: CXCL9, CXCL10, and CXCL11.

## Materials and methods

### Study design and participants

This cross-sectional study was approved by the Region Zealand Ethics Committee, Denmark, and adhered to the Helsinki declaration’s tenets. All participants provided written and oral informed consent. The study was performed at the ophthalmology- and hematology departments, Zealand University Hospital, Roskilde. Patients in the outpatient programs in the two departments were asked to participate. The participants consisted of 30 patients with nAMD, 30 iAMD (Beckmann Classification) [2], and 63 MPNs (WHO2016 criteria) [22]; of those 35 MPNs with early or intermediate AMD (MPNd) and 28 with normal retinas (MPNn). Inclusion was done between July 2018 and November 2020 [23, 24]. Exclusion criteria were patients having other concurrent cancer, inflammatory- or autoimmune diseases. Patients were also excluded if receiving immunomodulating therapy (Ruxolitinib, interferon-a), recent anti-VEGF therapy for nAMD (within the last eight weeks), or having a CRP greater than 15. Four patients were excluded post hoc, one nAMD (CRP>15), two iAMD (fulfilled GA criteria), and one MPNn (flow cytometric analyses failed).

## Retinal imaging and clinical data

We obtained stereoscopic 45° color fundus photographs centered on the macula (model TRG-NW8, Topcon). We diagnosed nAMD according to standard procedures (OCT and angiography). Pupil dilation was done before photographing (tropicamide 1%). All participants were subjected to a questionnaire about their health status, medication, and lifestyle.

### Blood sampling, flow cytometry, and immunoassays

The same investigator (C.L.) drew blood from antecubital veins in ethylene-diamine-tetraacetic-acid-coated (EDTA) tubes for flow cytometry and lithium-heparin-coated tubes for CRP analysis and immunoassays. Blood used to isolate plasma was centrifuged, and the plasma was immediately stored frozen (-80°C) for later immunoassay analyses.

**Fig 1:**
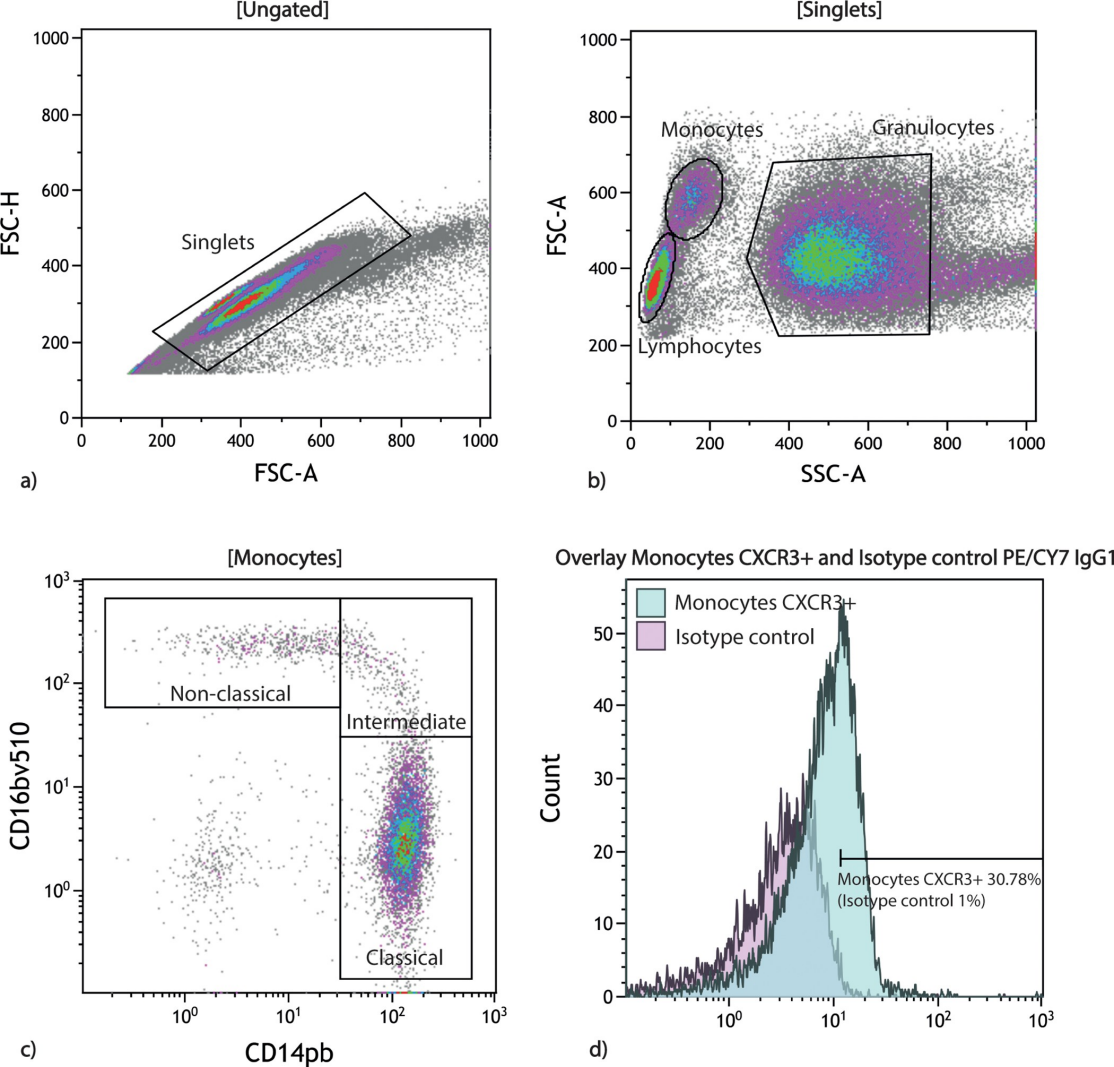
Gating strategy for identifying CXCR3 expression on monocytes using Kaluza software. a) First, we identified Singlets using forward height vs. area scatter. b) Next, we identified monocytes using a forward side scatter plot. c) Monocyte subsets were identified using the markers CD14 and CD16 (classical monocytes CD14^+^CD16^-^, intermediate monocytes CD14^+^CD16^+^, and non-classical monocytes CD14^-^CD16^+^). d) Finally, we used a negative isotype control (set to 1%) to distinguish the CXCR3 positive monocytes (shown in the overlay plot). The same procedure was done for lymphocytes, using the markers CD4+ and CD8+ for identifying CD4+ and CD8+ T cells.

We performed flow cytometric analyses within four hours. A Sysmex KX-21NTM (Sysmex Corporation) measured white blood count, and from this count, we calculated the blood needed to obtain 1.0 x 10^6^ white blood cells in the test tube. We used a 1% lysis buffer (Nordic BioSite AB) to lyse erythrocytes. We washed the cells in BD FACS Flow isotonic buffer (BD Biosciences) and centrifuged the cells at 500xg for 5 min. We repeated the washing/centrifuging process additional two times. Cells were resuspended in isotonic buffer, and we added monoclonal antibodies, leaving them to incubate for 20min in the dark at room temperature. We used one flow cytometry panel for monocytes and one for T cells, and we used corresponding fluorochrome-matched isotype controls. In the T cell panel, we used Brilliant Violet V510 CD8 IgG1κ (BioLegend, 301048), Phycoerythrin (PE)/Cyanine7(Cy7) CXCR3 IgG1 (BD biosciences, 560831), Peridinin-chlorophyll-protein(PerCP) CD4 IgG2a (R&D Systems, FAB3791C). In the monocyte panel, we used Brilliant Violet V510 CD16 IgG1κ (BioLegend, 302048), PE/Cy7 CXCR3 IgG1 (BD biosciences, 560831), Pacific Blue CD14 IgG1κ (BioLegend, 325616). The following isotype controls from BioLegend were used: Brilliant Violet V510 IgG1 (400172), PE/Cy7 IgG1 (400126), Pacific Blue IgG1 (400151), PerCP IgG2a (400250). In the final preparation step, we washed and resuspended the stained cells in isotonic buffer and analyzed the cells on a BD FACSCantoII flow cytometer (BD Biosciences) with a gating size of 100,000 singlet leukocytes. We analyzed the flow data with Kaluza Analysis software (v. 2.1; Beckman Coulter). Fig 1 shows an example of the gating strategy.

Immunoassays were carried out at the Technical University of Denmark (DTU). According to the manufacturer’s instructions, chemokines were quantified with multiplex immunoassays from Meso Scale Discovery. We ran the tests in duplicates and determined the mean concentration and the coefficient of variation (CV). The mean CV values were between 1.9–3.6. The plates were read on a QuickPlex SQ120 (Meso Scale Discovery).

### Statistics

Data statistics were done with RStudio v.4.1.1. We based our power calculation on prior comparable immunologic studies of these patients, resulting in a sample size of a minimum of 26 in each group [23, 24].

We report normally distributed data as mean and 95% confidence interval (CI), and non-normal data as median and interquartile range (IQR). Normality was assessed with histograms and QQ-plots. Age dependency and correction were assessed with linear regression or robust linear regression. Multiple regressions were used to investigate effects on outcomes for variables in Table 1. For group comparisons, we used the independent samples t-test, Wilcoxon’s rank-sum test, One-way analysis of variance (ANOVA), or Kruskal Wallis test for continuous variables. We used the Chi-squared test or Fisher’s Exact test for categorical variables. P-values less than 5% were considered statistically significant. We did not use Bonferroni corrections since the comparisons were part of our preplanned hypotheses.

**Table 1:** Patient characteristics.

	nAMD (n = 29)	iAMD (n = 28)	MPNd (n = 35)	MPNn (n = 27)	p-value
**Demographics**					
Age, years, median (IQR)	77 (71–82)	73 (68–76)	72 (65–76)	69 (62–74)	**<0.001** *
Sex					0.28^†^
*Males*, *n (%)*	12 (41)	10 (36)	20 (57)	10 (37)	
*Females*, *n (%)*	17 (59)	18 (64)	15 (43)	17 (63)	
**Lifestyle factors**					
Smoking, n (%)					0.83^‡^
*Never*	12 (41)	12 (43)	16 (46)	11 (41)	
*Former*	13 (45)	13 (46)	18 (51)	14 (52)	
*Current*	4 (14)	3 (11)	1 (3)	2 (7)	
Body mass index, mean (95%CI)	26 (24–27)	25 (24–27)	25 (24–27)	27 (25–29)	0.48^§^
Alcohol consumption, units per week, median (IQR)	2 (0–7)	3 (0–7)	7 (2–14)	2 (0–8)	**0.0036** *
**Comorbidities**					
Cardiovascular disease, n (%)	4 (14)	5 (18)	6 (17)	6 (22)	0.89^c^
Hypertension, n (%)	13 (45)	8 (29)	18 (51)	17 (63)	0.075^†^
Hypercholesterolemia, n (%)	4 (14)	2 (7)	3 (9)	2 (7)	0.82^‡^
Type 2 diabetes, n (%)	2(7)	1 (4)	2 (6)	0 (0)	0.76^‡^

Significant p-values are shown in bold. Statistical comparisons between groups:

*Kruskal Wallis test

^†^Pearson’s Chi-squared test

^‡^Fischer’s exact test

^§^One-way ANOVA. AMD: age-related macular degeneration, nAMD: neovascular AMD, iAMD: intermediate AMD, MPN: myeloproliferative neoplasms, MPNd: Patients with MPN and drusen, MPNn: patients with MPN and normal retinas, IQR: interquartile range.

## Results

### Study population

Table 1 shows patient characteristics. The median age in nAMD was 77 years (IQR:71–82), significantly older than the three other groups; iAMD 73 (IQR:68–76), p = 0.034; MPNd 72 (IQR:65–76), p = 0.0040; and MPNn 69 (IQR: 62–74) p<0.001. We found no difference in sex, body mass index, smoking habits, or comorbidities between the groups. The median alcohol consumption in MPNd was seven units per week, significantly higher than nAMD with 2 (IQR:0–7), p<0.001; iAMD 3 (IQR:0–7), p = 0.019, and MPNn 2 (IQR:0–8), p = 0.021. All outcomes described below were corrected for age where appropriate and alcohol consumption or other patient characteristics mentioned above did not influence outcomes reported in this work. The MPNs consisted of 39 PV-, 17 ET-, and six MF patients, and 53 (85%) had the *JAK2V617F*-mutation, four (6.2%) *CALR*, and one (1.5%) *MPL*. The median *JAK2V617F* allele burden was 33% (IQR:11–56) in MPNd and 17% (IQR:5.6–28) in MPNn (p = 0.088). Patients receiving hydroxyurea were similar in the MPN groups (p = 0.99), and all MPN received acetylsalicylic acid or other anticoagulant therapy. Patients receiving statins were similar across all groups (p-value = 0.58).

### Leukocyte CXCR3

CXCR3 expression was not dependent on age on CD4+ T cells and monocytes and subsets but decreased with age on CD8+ T cells. For CD4+ T cells and monocytes, we used the Kruskal Wallis test and Wilcoxon rank-sum test for comparisons between groups, and we used robust linear regression to compare groups for CD8+ T Cells.

**Fig 2:**
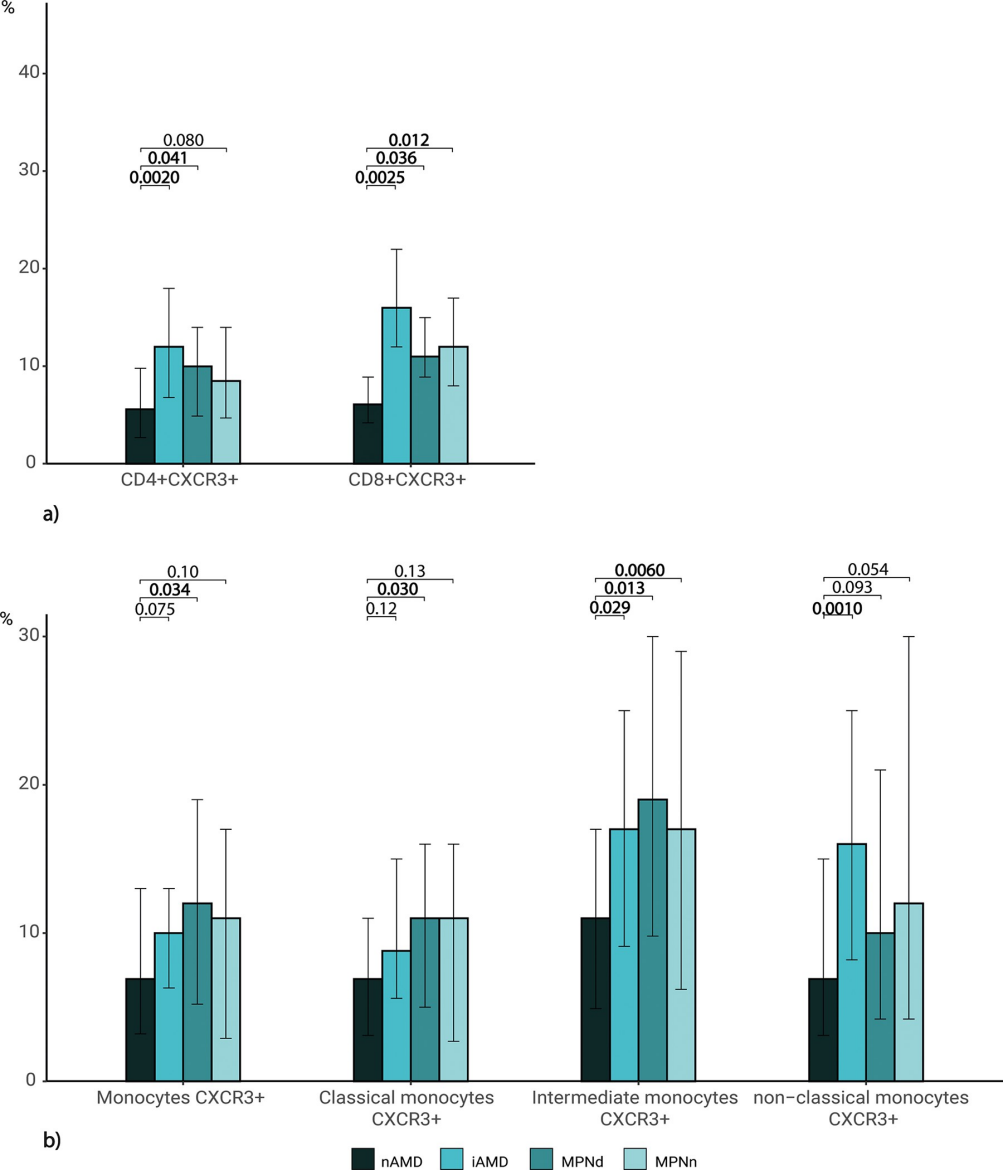
Lower CXCR3 expression on leukocytes in patients with nAMD. Median frequency (%) of leukocytes and leukocytes expressing CXCR3 in patients with neovascular AMD (nAMD), intermediate AMD (iAMD), myeloproliferative neoplasms with drusen (MPNd), and myeloproliferative neoplasms with normal retinas (MPNn) **a)** Expression of CXCR3 on T cells. **b)** Expression of CXCR3 on monocytes. Bars represent the interquartile range. Statistical comparison between groups: Kruskal Wallis or if the outcome depended on age, robust linear regression. Wilcoxon rank-sum test for multiple comparisons. Significant P-values are shown in bold.

The CD8+ T cell CXCR3 expression in patients with nAMD was 6.1% (4.2–8.9) significantly lower than the other groups (iAMD 16% (12–22) p = 0.0025, MPNd 11% (8.9–15) p = 0.036, MPNn 12% (8.0–17) p = 0.012) (Fig 2A). A lower CD4+CXCR3 expression was also observed when comparing nAMD to the other groups, except for MPNn (p = 0.080) (Fig 2A). The intermediate monocyte CXCR3 expression in nAMD was 11% (4.9–17), significantly lower than 17% (9.1–25) in iAMD (p = 0.029), 19% (9.8–30) in MPNd (p = 0.013) and 17% (6.2–29) in MPNn (p = 0.0060). The same tendency was seen in monocytes, classical- and non-classical monocytes with significantly lower levels in nAMD than in some of the other groups (Fig 2B). No significant differences were found between MPNd and MPNn (Fig 2).

**Fig 3:**
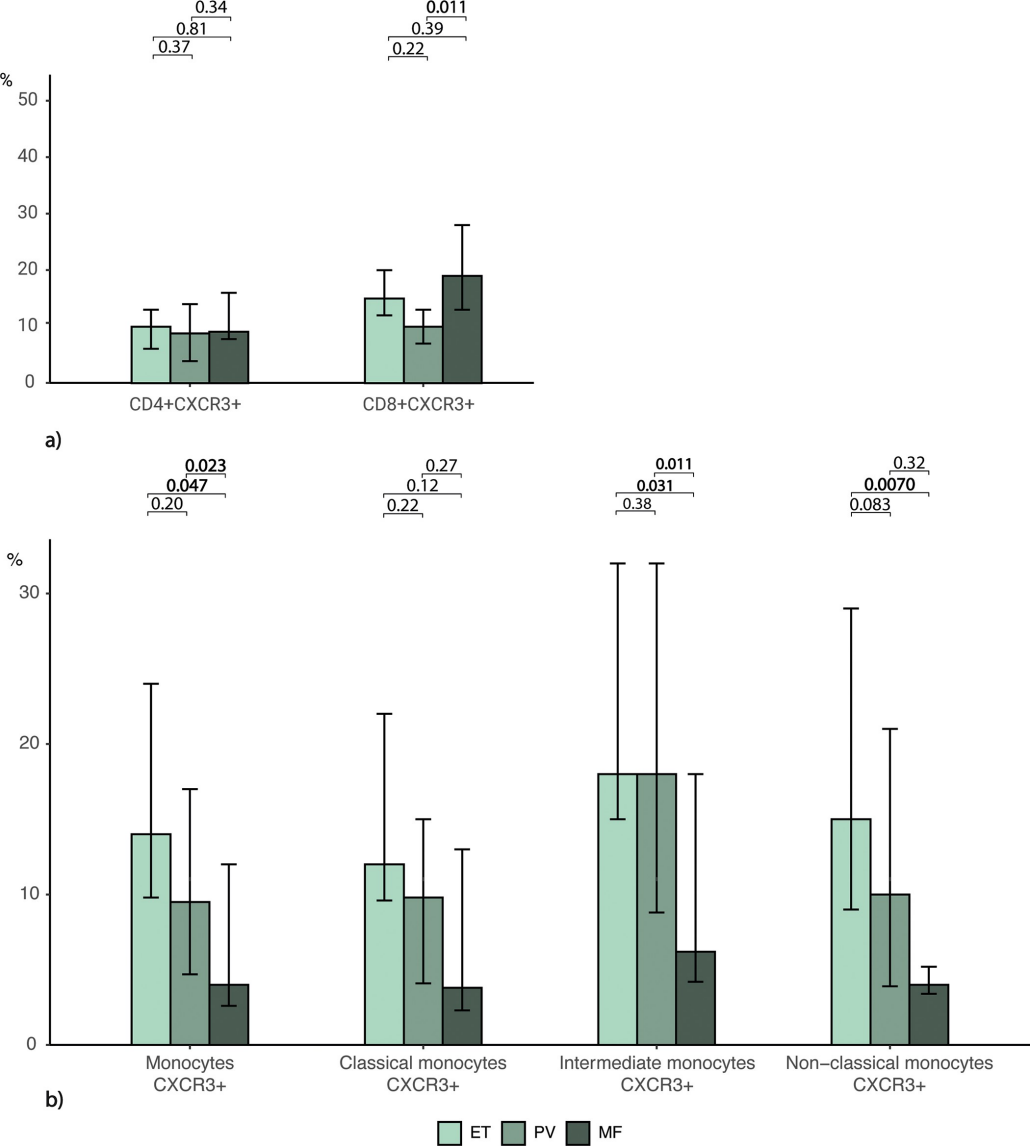
CXCR3 expression on leukocytes in MPN subtypes showing decreasing levels of CXCR3 on monocytes over the MPN biological continuum in monocytes. Median frequency (%) of leukocytes and leukocytes expressing CXCR3 in patients with essential thrombocythemia (ET), polycythemia vera (PV), and myelofibrosis (MF) **a)** Expression of CXCR3 on T cells. **b)** Expression of CXCR3 on monocytes. Bars represent the interquartile range. Statistical comparison between groups: Kruskal Wallis or if the outcome depended on age, robust linear regression. Wilcoxon rank-sum test for multiple comparisons. Significant P-values are shown in bold.

When we investigated the MPN biological continuum (Fig 3), we found CXCR3 expression on CD8+ T cells significantly lower in PV with 11% (5.9–20) than in MF with 17% (15–24) (p = 0.011) but not than in ET with 15% (11–22) (p = 0.22). There was no difference between MPN subtypes on CD4+ T cells (p = 0.48) (Fig 3A). Fig 3B shows the CXCR3 expression on monocytes over the biological continuum. The expression on overall-monocytes and intermediate- and non-classical monocytes was in MF 4.0% (2.6–12), 6.2% (4.2–18) and 4.0 (3.4–5.2), significantly lower compared to ET with 14% (9.8–24), 18% (15–32) and 15% (9.0–29) (p = 0.047, p = 0.031, p = 0.0070, respectively). On overall monocytes and intermediate monocytes, the CXCR3 expression was also significantly lower in MF than PV with 9.5% (4.7–17) and 18% (8.8–32) (p = 0.023, p = 0.011). There was an overall tendency for decreasing levels across the biological continuum.

We also investigated leukocyte percentages (Table 2). Patients with iAMD had a significantly higher lymphocyte percentage of 16% (CI:14–19) than the other groups (nAMD 11% (CI9.3–14) p = 0.040, MPNd 9.4% (CI:8.0–11) p<0.001, MPNn 10% (CI8.8–12) p<0.001). We also observed an intermediate monocyte percentage of 9.2% (CI:7.5–11) in MPNd, significantly higher than 6.3% (CI:4.7–8.5) in nAMD (p = 0.037) and 6.3% (CI: 5.2–7.6) iAMD (p = 0.0060). We found no differences in leukocyte percentages between MPN subtypes except for the lymphocyte percentage, which was higher in ET with 13% (CI:10–15) than PV with 9.3% (CI:8.0–11) (p = 0.050) and MF with 7.1% (CI:5.8–8.7) (p<0.001) (S1 File).

**Table 2:** Mean percentages of leukocytes in patients with nAMD, iAMD, MPNd, and MPNn.

	nAMD (n = 29)	iAMD (n = 28)	MPNd (n = 35)	MPNn (n = 27)	p-value
Lymphocytes %, mean (95% CI)	11 (9.3–14)	16 (14–19)	9.4 (8.0–11)	10 (8.8–12)	**<0.001** *
CD4+, %, mean (95% CI)	40 (36–43)	42 (38–45)	40 (35–44)	40 (35–45)	0.81^†^
CD8+, %, mean (95% CI)	23 (20–26)	21 (18–24)	18 (15–21)	19 (16–23)	0.15*
CD4/CD8 ratio mean (95% CI)	1.7 (1.4–2.1)	1.9 (1.6–2.4)	2.1 (1.6–2.6)	2.0 (1.6–2.6)	0.61*
Monocytes %, mean (95% CI)	7.2 (6.1–8.1)	6.9 (6.1–19)	6.7 (5.5–12)	6.2 (5.4–17)	0.64*
Classical monocytes, %, mean (95% CI)	85 (82–88)	84 (82–86)	80 (76–83)	81 (76–86)	0.093*
Intermediate monocytes, %, mean (95% CI)	6.3 (4.7–8.5)	6.3 (5.2–7.6)	9.2 (7.5–11)	8.2 (6.2–11)	**0.049** *
Non-classical monocytes %, mean (95% CI)	6.7 (4.7–9.5)	7.1 (6.5–8.4)	7.1 (5.6–8.9)	6.6 (5.2–8.2)	0.37^‡^

*one-way ANOVA

^†^Welch ANOVA

^‡^Two-way ANOVA

95% CI: 95% confidence interval. nAMD: neovascular AMD, iAMD: intermediate AMD, MPNd: myeloproliferative neoplasms with drusen, MPNn: myeloproliferative neoplasms with normal retinas.

### Plasma CXCL9/-10/-11

The CXCL9- and CXCL10 levels increased with age (both p<0.001), and we, therefore, used robust linear regression to compare groups for these outcomes. We found no differences in the levels of CXCL9 and CXCL10 between any of the groups (Table 3). The levels of CXCL11 were not age-dependent, and we used the Kruskal Wallis test and Wilcoxon rank-sum test to compare groups. We found higher levels in both MPN groups than the AMD groups (nAMD-MPNd: p = 0.0050, nAMD-MPNn: p = 0.0028, iAMD-MPNd: p = 0.0026, iAMD-MPNn: p = 0.0035). There were no differences between subtypes of MPNs (ET, PV, and MF) in levels of CXCL9, CXCL10, and CXCL11 (Data not shown).

**Table 3:** Median plasma concentration of CXCL9, CXCL10, and CXCL11.

	nAMD (n = 29)	iAMD (n = 28)	MPNd (n = 35)	MPNn (n = 27)	P-value between all groups	P-value between MPN groups
CXCL9 pg/ml (IQR)	84 (52–117)	52(43–103)	64 (47–77)	54 (41–65)	0.41*	0.38*
CXCL10 pg/ml (IQR)	750 (659–1283)	685 (576–941)	786 (639–1163)	740 (565–1020)	0.44*	0.78*
CXCL11 pg/ml (IQR)	739 (637–976)	680 (594–1089)	1118 (794–1851)	992 (870–1752)	**0.00069** ^†^	0.81^‡^

*robust linear regression

^†^Kruskal Wallis test

^‡^Wilcoxon rank-sum test

IQR: interquartile range. nAMD: neovascular AMD, iAMD: intermediate AMD, MPNd: myeloproliferative neoplasms with drusen, MPNn: myeloproliferative neoplasms with normal retinas.

## Discussion

This study aimed to investigate leukocyte CXCR3 expression in patients with nAMD, iAMD, MPNd, and MPNn. The CXCR3 expression is interesting since it has been strongly related to angiogenesis- and fibrosis inhibition, and it is found to be lower in nAMD than in healthy controls. We wanted to investigate if this lower expression in nAMD was also seen when compared to iAMD. We also speculated if we could find a low CXCR3 expression over the MPN biological continuum, especially in the MPN subtype MF, which shows excessive bone marrow angiogenesis and fibrosis, as patients with nAMD show in the retina.

### CXCR3

We found a lower CXCR3 expression in patients with nAMD than iAMD, MPNd, and MPNn, in CD4+ and CD8+ T cells and a tendency for a lower expression on all monocyte subsets, with a significantly lower expression on intermediate- and non-classical monocytes.

Our group previously found lower CXCR3 expression on peripheral CD8+ T cells in patients with nAMD than in a healthy control group, indicating a systemic CXCR3 dysregulation [15]. This study also finds a lower CXCR3 expression when comparing nAMD and iAMD. It would be interesting to investigate if we could find differences between iAMD and healthy controls.

The CXCR3-axis has various functions, such as regulating immune cell migration, differentiation, and activation [25, 26], and is expressed on numerous cells throughout the body, including leukocytes and endothelial cells [27].

The binding of CXCL9/10/11 to CXCR3 is known to have angiostatic and antifibrotic properties [28, 29]. For instance, CXCL10 binding to CXCR3 has been shown to reduce endothelial cell migration and the ability of these cells to form tubes [30]. CXCR3 is expressed in both human and mouse choroidal endothelial cells. A study found increased CXCL10 and CXCR3 expression in CNV tissue from mice with laser-induced CNVs. The same study found that CXCR3-deficient mice developed larger laser-induced CNV with more fluid leakage and macrophage infiltration than wild-type mice [18]. Other experimental studies on endothelial cells show that binding of CXCL10 to CXCR3 can have a counterproductive effect on VEGF stimulation of endothelial cells and decreases this activity, and further blocking of CXCR3 results in CXCL10 not being able to override the VEGF signal, which leads to angiogenesis [30, 31].

When we compared the CXCR3 expression in MPNs, we found no differences between MPNd and MPNn. However, when we investigated the biological continuum, a tendency of a lower expression across the continuum from ET/PV to MF was observed in all monocyte subsets, with significant differences between subgroups in intermediate- and non-classical monocytes. It seems that advanced stages of MPNs are also associated with a lower CXCR3 expression. Interestingly, angiogenesis and fibrosis are seen in later stages of MPN (especially MF) and in nAMD, and the two conditions simultaneously show a CXCR3 downregulation.

The CXCR3 receptor has three alternative splice variants which activate different intracellular signaling pathways. Which of the intracellular pathways are activated depends on the receptor, the binding ligand, and the type of cell or tissue studied, a phenomenon called “biased signaling” or “biased agonism”. Biased signaling adds complexity to the chemokine-receptor axis [32, 33]. Therefore, the three CXCR3 ligands are also thought to have different roles in T cell trafficking. CXCL9 and -10 seem to induce inflammation by Th1/Th17 cell polarization and recruitment, while CXCL11, which binds CXCR3 with the highest affinity, appears to induce T cell polarization into Th2 and regulatory T cells and can thereby play a role in restricting inflammation [34]. Thus, the ligand-CXCR3 axis may have opposing effects on regulating the biological T cell function. The CXCR3 expression on many different cells links cell-mediated immunity and angiogenesis. In addition to the direct effect of CXCR3 signaling on endothelial cells, the IFN-γ inducible chemokines CXCL9 and -10 induce a Th-1 type immune response by attracting CXCR3 expressing T cells (Th1 T cells), monocytes, and natural killer cells, which lead to the production of more IFN-γ, inducing increased expression of the IFN-γ inducible chemokines, which again can recruit and activate more CXCR3 expressing cells. This process of type 1 immune response occurring along with inhibition of angiogenesis (immunoangiostasis) in a positive feedback loop seems, from our results, dysregulated (lower) in nAMD and also in MPN with more advanced disease [6, 35, 36].

The systemic CXCR3 alterations could affect the retina. A study has shown that monocytes and endothelial cells can directly interact and synergistically induce the expression of CXCL10 [36]. Macrophages play an important role in immune regulation and pathogenic angiogenesis [37, 38], including the neovascularization seen in nAMD [37, 39, 40]. Tissue macrophages derive from blood monocytes, which can be classified as classical- (CD14^+^CD16^-^), intermediate (CD14^+^CD16^+^), and non-classical monocytes (CD14^-^CD16^+^). In particular, the intermediate monocytes, and especially those expressing the angiopoietin-2 receptor, Tie-2, have been characterized as highly pro-angiogenic cells, which secrete pro-angiogenic factors such as VEGF [41, 42]. Compelling evidence suggests that blood-derived monocytes can enter the subretinal space, and changes in monocytes’ expression of different receptors could influence the subretinal milieu [43–45].

In addition to monocytes being able to cross the blood-retina-barrier, lymphocytes have been observed in the choroid of eyes from AMD patients [46–48] and CD8 positive cells seem present to a higher degree in the macular choroid of patients with drusen [48].

We can only guess what the lower CXCR3 expression in nAMD and advanced MPN means. However, we may suggest that the low expression could result in altered T helper cell polarization and/or an imbalance between angiogenic and angiostatic factors and accompanying dysregulated angiogenesis (dysfunctional immunoangiostasis).

### CXCL9/10/11

When we investigated the IFN-γ inducible chemokines, we found no differences in CXCL9 and -10 levels between groups, but both MPN groups had higher levels of CXCL11 than the two AMD groups. This could be interesting to investigate further in future studies. One hypothesis could be that the higher CXCL11 levels could be a compensatory mechanism for the high level of chronic inflammation seen with these diseases since, as mentioned, CXCL11 could play a part in restricting inflammation. There are limited in-vivo studies of CXCL11 functions, but studies have found elevated levels in patients with autoimmune disease [49, 50] and high levels of all three IFN-γ inducible chemokines have been associated with a poor prognosis in many types of cancers [51].

Many studies have investigated cytokine levels in the aqueous humor of patients with nAMD, and several cytokines have been found elevated, including CXCL9 and -10 [52, 53]. Further, levels of CXCL-10 and -11 in post-mortem eyes have been found to correlate with the presence of drusen [54]. Only a few studies have investigated serum/plasma levels of the IFN-γ inducible chemokines in patients with nAMD. One study found no differences in plasma levels of these chemokines between a healthy age-matched control group and patients with early AMD, nAMD, and geographic atrophy [15]. Another study found CXCL10 levels elevated in the serum of all AMD stages compared to healthy controls [17]. This study found the highest levels in intermediate AMD and a slight decrease in nAMD. The same study investigated donor’s eyes and found the CXCL10 expression increased in the RPE and is expressed by neovascular endothelial cells.

### Limitations

Several limitations must be kept in mind when interpreting this study’s results. The study is only observational, and further experimental studies are needed to infer causality. The groups compared are also small, especially the number of patients with MF. It is also worth remembering that the chemokines and cells discussed in this paper are part of a large, complex, and interacting system, and we are looking at a few isolated systemic differences.

## Conclusions

We find a CXCR3 downregulation on T-cells and some monocyte subset in patients with nAMD compared to iAMD, MPNd, and MPNn, in line with previous nAMD studies [15, 16]. We also find a CXCR3 downregulation in most monocyte subsets over the MPN continuum, a disease group having increased drusen/AMD prevalence. The advanced MF stage has characteristics like nAMD with angiogenesis and fibrosis and, at the same time, low CXCR3 expression. Therefore, systemically leukocyte CXCR3 expression could both be involved in changes seen in the retina and the bone marrow.

The principal nAMD feature is CNV formation. Angiogenesis regulating mechanisms are complex, but VEGF is a dominant stimulator, and AMD is treated with anti-VEGF eye injections, which have proven effective. However, many patients still lose vision despite treatment, and the need for new targets and treatment is evident, especially therapy that can be initiated earlier in the disease progresses. Our findings could support a role of CXCR3 in AMD pathogenesis, and further understanding the CXCR3-axis and immune cell trafficking in AMD may elucidate underlying pathogenic mechanisms and reveal new treatment targets. Targeting chemokines or their receptors may be an effective therapeutic approach, as suggested for other inflammatory and neoplastic diseases [55–58]. Since chemokines have cross-talk with other angiogenic factors such as VEGF, combined targeting of VEGF and chemokines may have synergistic effects.

## Supporting information

S1 File (XLSX)
